# Optimization of Deep Eutectic Solvent-Based Ultrasound-Assisted Extraction of Bioactive Compounds from Maca Leaves Using the Taguchi Method

**DOI:** 10.3390/molecules30071635

**Published:** 2025-04-06

**Authors:** Eun Ji Lee, Kyung Young Yoon

**Affiliations:** Department of Food and Nutrition, Yeungnam University, Gyeongsan 38541, Republic of Korea

**Keywords:** maca leaves, deep eutectic solvent, ultrasound-assisted extraction, saponin, polyphenol, Taguchi method

## Abstract

This study was conducted to identify the optimal conditions and evaluate the feasibility of deep eutectic solvent (DES)-based ultrasound-assisted extraction (UAE) for utilizing maca (*Lepidium meyenii*) leaves, an agricultural by-product, as functional materials. The extraction parameters influencing the recovery of saponins and polyphenols, which are major bioactive compounds, were analyzed using the Taguchi method. Results: Signal-to-noise ratios and analysis of variance indicated that the liquid–solid ratio was the most critical factor for optimizing the extraction process. The optimal extraction conditions were determined to be a liquid–solid ratio of 40 mL/g, a water content in DES of 30%, an extraction time of 30 min, and an ultrasonic power of 300 W in the DES system consisting of choline chloride and glycerin in the molar ratio of 1:2. Maca leaf extract obtained under optimized DES-based UAE conditions exhibited higher bioactive compounds content and antioxidant activity compared with that obtained by hot water extraction. Therefore, the DES-based UAE method is a promising, eco-friendly alternative for extracting bioactive compounds from maca leaves.

## 1. Introduction

Maca (*Lepidium meyenii*), also known as Peruvian ginseng, is a cruciferous crop that grows in the Andes region of South America. Maca is hardy, can grow in inhospitable natural environments, and is resistant to strong winds, cold, and drought [1]. Maca roots contain polyphenols, tannins, saponins, steroids, and a variety of organic compounds, including macaenes, macamides, glucosinolates, isothiocyanates, and alkaloids [2]. These components have been reported to act alone or synergistically to exhibit biological activities, which include neuro and liver protection, antioxidant, anti-fatigue, anti-cancer, and anti-osteoporosis properties, and immune regulation [3]. Accordingly, the consumption of maca root is continuously increasing; however, the maca leaves, a by-product generated during maca processing, are discarded as waste [4]. According to recent study on the bioactive compounds and biological activities of methanol extracts of maca leaves, they have a higher content of saponins, phenols, and flavonoids than maca root extracts [5]. In addition, the leaves contain various polyphenols, such as kuwanone-G, oxypaenoniflorin, laricitrin, and ligustroflavone [5,6]. Moreover, the aerial parts of maca plants (leaf, stem, and inflorescence) contain glucosinolates, macamides, and saponins at ranges of 31.4–36.2, 0.451–0.703, and 33.2–51.8, mg/g (dry weight), respectively, at different growth stages [7]. However, although maca leaves contain a variety of bioactive compounds, they are currently not viewed as a potential source of functional substances [7]. Therefore, research is needed to explore the potential of maca leaves discarded as waste as a functional material.

To recover bioactive substances from a plant matrix, an appropriate extraction process is required. Recently, eco-friendly extraction technologies, such as supercritical fluid extraction, ultrasound-assisted extraction (UAE), and microwave-assisted extraction (MAE) have been applied to replace conventional extraction methods [8]. Among these technologies, UAE is widely used, where ultrasound can improve the mass transfer of compounds from plant substrates to solvents by generating cavitation bubbles that disrupt the plant cell walls. This improves the extraction yield and saves time, energy, and solvents compared to conventional extraction methods [9]. In addition, interest in the use of eco-friendly solvents that do not negatively impact the environment and humans is increasing; among these, deep eutectic solvent (DES), which has low toxicity, is the most promising [10]. A DES can be simply prepared as an eutectic mixture containing a mixture of a hydrogen bond donor and acceptor molecules. That is, when two chemicals are stirred together at a specific temperature, they mutually induce melting and form hydrogen bonds, resulting in a transparent solution of DES at a temperature significantly lower than the melting points of the individual substances [11]. Utilizing DES as an extraction solvent facilitates the isolation of target components from plant materials through hydrogen bonding and electrostatic interactions between DES constituents and the target molecules [12]. DES offers several advantages, including simple production, low toxicity, environmental compatibility, high biocompatibility and biodegradability, and low cost. Furthermore, DES can be synthesized with diverse chemical properties by altering one or two of its components, making it a versatile solvent for the extraction of various compounds, thereby overcoming the limitations of existing extraction solvents [13]. In addition, DES can be efficiently used to extract various nonpolar and polar bioactive substances from natural plant resources by using a combination of thermal extraction, UAE, and MAE [14]. In particular, many studies have been conducted to extract various bioactive components such as polysaccharides and polyphenols from plants using DES-based UAE and to improve extraction efficiency [12,15,16]. However, no studies have been conducted to extract bioactive substances such as saponins and polyphenols from maca leaves.

Therefore, this study aimed to determine the optimal DES-based UAE conditions to improve the extraction efficiency of bioactive compounds, including polyphenols and saponins, from maca leaves. For this purpose, the extraction conditions, including the liquid–solid ratio, water content in DES, extraction time, and ultrasonic power, were optimized using Taguchi’s methodology. In addition, the bioactive compounds and antioxidant activities of the maca leaf extracts obtained under the optimized DES-based UAE conditions were compared with those from hot water extraction (HWE), one of the representative conventional extraction methods.

## 2. Results and Discussion

### 2.1. Total Saponin and Polyphenol Contents

To determine the optimal conditions for DES-based UAE for the maximum recovery of bioactive compounds from maca leaves, four factors (liquid–solid ratio, water content in DES, extraction time, and ultrasonic power) were set to three levels, and sample extraction was performed according to the L_9_ (3^4^) orthogonal matrix. The results of orthogonal experiments are shown in Table 1. The total saponin content (TSC) of the extracts obtained under various conditions was 16.43–33.43 mg oleanolic acid equivalent (OAE)/g dry weight (DW); the highest content of extracted TSC was achieved using an liquid–solid ratio of 40 mL/g, 30% water content, 20 min extraction time, and 180 W ultrasonic power. The total polyphenol content (TPC) obtained according to each parameter was found to be 3.10–6.32 mg gallic acid equivalent (GAE)/g DW, and runs 7–9 showed higher TPC. For runs 7–9, the water content, extraction time, and ultrasonic power showed some differences, but the liquid–solid ratio was the same at 40 mL/g. In addition, the TSC and TPC increased with the increasing liquid–solid ratio, and the highest TSC and TPC were obtained at a liquid–solid ratio of 40 mL/g, which is consistent with the results reported by Liu et al. [17].

The liquid–solid ratio is reported to substantially impact the solvent consumption, recovery of target compounds, and solvent regeneration costs. In general, as the liquid–solid ratio increases, the yield of phenolic compounds increases and the extraction time is shortened. This is because a larger volume of solvent increases the contact area with the target material, accelerating the diffusion of the solute into the solvent [18]. However, considering the solvent and target compound recovery, it is necessary to establish an optimal range that balances extraction yield and solvent consumption [19].

In general, the extraction ability of DES for bioactive substances is related to its physical and chemical properties, such as hydrogen bonding, viscosity, polarity, and pH [20]. In particular, adding water to DES, which is highly viscous, can lower its viscosity; however, this may increase its polarity and weaken the hydrogen bonds between acceptor and donor molecules [21,22]. Therefore, to promote the transfer of the target ingredient to the solvent, it is necessary to adjust the amount of water added, that is, the viscosity of DES [23]. The *K* value showed that the TSC and TPC increased as the water content of the DES increased. The highest TSC and TPC were observed at 30.43 mg OAE/g DW and 5.21 mg GAE/g DW, respectively, when the water content added to DES was 30%. Considering these results, the saponins and polyphenols extracted from the maca leaves using DES prepared with choline chloride and glycerin, which are polar substances, and the 30% water content in DES, are suggested to have increased the extraction yield by appropriately lowering the viscosity and increasing the polarity of the solvent.

### 2.2. Analysis of Main Effect on Bioactive Compounds Recovery

To examine the effect of extraction conditions on the recovery of the bioactive compounds in the maca leaf extract, the effect of each factor was calculated based on an orthogonal test (Table 1). The delta value represents the relative effect of each factor on the response as the difference between the maximum and minimum average response values for each factor. Based on the average delta value for the responses obtained at each factor-variable level in this study, the TSC was influenced by the parameters in the following order: liquid–solid ratio (9.17 mg OAE/g DW), water content (7.39 mg OAE/g DW), ultrasonic power (1.56 mg OAE/g DW), and extraction time (2.20 mg OAE/g DW). Similarly, the TPC was affected by the parameters in the following order: liquid–solid ratio (2.68 mg GAE/g DW), water content (0.57 mg GAE/g DW), ultrasonic power (0.28 mg GAE/g DW), and extraction time (0.51 mg GAE/g DW).

In the Taguchi method, the experimental conditions with the least variability are identified as the optimal conditions, and this variability is expressed as the signal-to-noise (S/N) ratio. Since variable characteristics are inversely proportional to the S/N ratio, the experimental conditions with the highest S/N ratios are considered optimal [24]. Additionally, depending on the objective of the experiment, the results can be interpreted using the most appropriate method between nominal-is-best, smaller-the-best, and larger-the-best characteristics [18]. As shown in Table 1, in this study, the TSC showed the highest S/N ratio at run 9 (30.37), and the TPC showed the highest S/N ratio at runs 8 and 9 (15.99).

**Figure 1:**
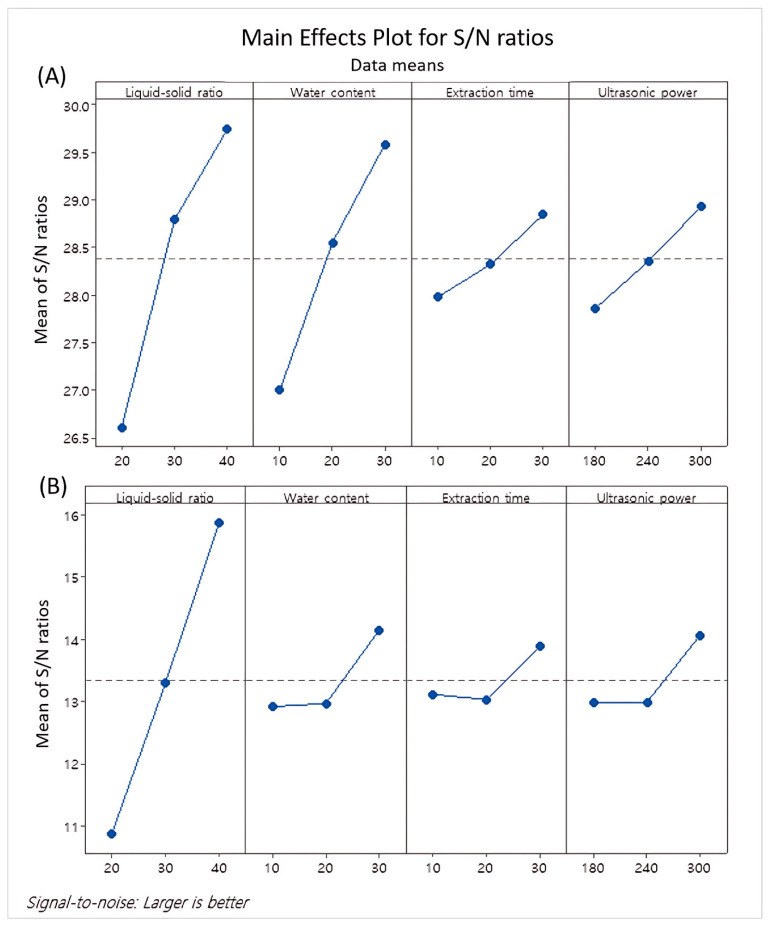
Main effects plot for mean of total saponin content (**A**) and total polyphenol content (**B**).

In the main effect plot for the S/N ratios, the mean S/N ratios for each factor were plotted against the test levels for each factor parameter (Figure 1). The main effects plot of TSC indicates that the liquid–solid ratio had the largest influence, followed by the water content, while the extraction time and ultrasonic power had very slight impacts (Figure 1A). Similarly, the TPC was greatly influenced by the liquid–solid ratio, with the effects of the other parameters being very slight (Figure 1B).

### 2.3. Analysis of Variance (ANOVA) Results

The Taguchi experimental design uses orthogonal arrays and ANOVA as analysis tools. After conducting a minimal number of experiments using the orthogonal array method, the effect of the specific factors studied on the response is measured using ANOVA. The ANOVA was conducted at a 95% confidence level to identify the significant variables and quantify their effects on the responses (Table 2). The ANOVA results indicated that all the selected factors were substantial parameters of the DES-based UAE process at the 95% confidence level (*p* < 0.05). Considering the percentage contribution of each factor on extraction, the results revealed that the liquid–solid ratio had the most influence on the TSC and TPC among the process parameters, probably owing to its highest percentage contribution and low *p*-value (*p* < 0.001). The liquid–solid ratio exhibited the highest percentage contribution (51.85%; *p* < 0.001) on the extracted TSC, followed by the water content (33.06%; *p* < 0.001), ultrasonic power (3.08%; *p* = 0.003), and extraction time (1.58%; *p* = 0.042). In the case of the TPC, the percentage contribution in the extraction process was in the same order as for the TSC; however, the liquid–solid ratio was found to have a higher impact on the TPC compared with the TSC, that is, the liquid–solid ratio had the highest contribution (81.95%; *p* < 0.001), followed by water content (4.76%; *p* < 0.001) and ultrasonic power (3.79%; *p* < 0.001). In contrast, the extraction time had little influence on the TPC (*p* > 0.05). Zeng et al. [15] studied the effects of the water content added to the DES, extraction temperature, and liquid–solid ratios on the extraction yield of polyphenols through response surface analysis, and found that the water content and the liquid–solid ratio had the greatest impact on the extraction yield; this is consistent with the results of this study. Based on analyzing the recovery of saponin and polyphenol using the Taguchi method in this study, the optimal responses were found to be as follows: liquid–solid ratio of 40 mL/g, 30% water content in DES, 30 min of extraction time, and 300 W of ultrasonic power.

Based on the experimental data presented in Table 1, linear regression analysis was performed to predict the relationship between the TSC or TPC and factors. The predicted equations, automatically formulated with Minitab 18 at a 95% confidence interval, were shown in Equations (1) and (2):

TSC = −0.25 + 0.4588A + 0.03695B + 0.0787C + 0.09839D(1)

TPC = −1.005 + 0.1338A + 0.0283B + 0.0117 + 0.00425D(2)

where A = liquid–solid ratio (mL/g), B = water content (%), C = extraction time (min), and D = ultrasonic power (W).

The *p* values of the derived regression equation for the TSC and TPC were 0.001 and 0.003, respectively, indicating the suitability of the proposed models (*R*^2^ = 98.24%, adjusted *R*^2^ = 96.48% for TSC; *R*^2^ = 96.89%, adjusted *R*^2^ = 93.78% for TPC).

### 2.4. Confirmation Test Results

In order to validate the predicted values derived from regression Equations (1) and (2) through the Taguchi method, the experimental values obtained under the optimal extraction conditions were measured and compared, and the results are presented in Table 3. Under optimal conditions, i.e., a liquid–solid ratio of 40 mL/g, water content of 30%, extraction time of 30 min, and ultrasonic power of 300 W, the predicted values for the TSC and TPC were 37.07 mg OAE/g DW and 6.82 mg GAE/g DW, respectively. The experimentally determined TSC and TPC values under the same extraction conditions were 38.02 mg OAE/g DW and 6.78 mg GAE/g DW, respectively. The differences between the predicted and experimental TSC and TPC values were minimal, at 0.95 and 0.04, respectively; this supported the notion that the regression model equations were suitably accurate within the 95% confidence interval, which ranged between 34.64 and 39.49 mg OAE/g DW for the TSC and 6.07 and 7.57 mg GAE/g DW for the TPC. Consequently, the close alignment between the experimental and predicted results, with the experimental values falling within the 95% confidence interval, suggests that the models are robust and reliable for predicting the TSC and TPC of the extracts obtained under optimal extraction conditions.

### 2.5. Comparison of Bioactive Properties of Maca Leaf Extracts

To gain insight into the superiority of DES-based UAE for the bioactive compounds of maca leaves, the TSC, TPC, and antioxidant activity of extracts obtained via DES-based UAE were compared with those of HWE, a conventional extraction method, and the results are shown in Table 4. The TSC of the extract obtained via DES-based UAE was 718.89 mg OAE/g and that via HWE was 707.50 mg OAE/g, which were much higher than those of Lee and Chang [5], who reported that the TSC of the methanol extract of maca leaves was 6.09 mg escin equivalents/100 g DW. In particular, this study confirmed that DES-based UAE was more effective than HWE in extracting saponins. These results are considered to be because saponins are polar compounds that are soluble in polar solvents such as water and alcohol, but insoluble in nonpolar organic solvents such as chloroform, ether, and acetone [25]. Yang et al. [26] reported that the extraction yield of physiologically active steroid saponins from extracts obtained from Dioscoreae nipponicae rhizoma using choline chloride and malonic acid as solvents was higher than that of water extracts, which was consistent with the results of this study.

The TPC of the maca leaf extracts obtained via DES-based UAE and HWE were 260.19 mg GAE/g and 224.90 mg GAE/g, indicating that DES-based UAE is more effective in extracting polyphenols. In addition, when measuring the content of phenolic compounds in the extracts, the extract obtained via DES-UAE showed a higher content than the extract obtained via HWE. For example, the phenolic compound content of the extract obtained via DES-based UAE was the highest in catechin at 102.58 mg/g, followed by gallic acid (17.82 mg/g), cinnamic acid (8.24 mg/g), and protocatechuic acid (6.92 mg/g). The extract obtained via HWE showed the highest in catechin at 88.73 mg/g, followed by gallic acid (15.77 mg/g), epicatechin (7.29 mg/g), and cinnamic acid (6.89 mg/g). Compared with conventional extraction solvents, the high viscosity of DES has a limitation: the extraction efficiency can be reduced because slow mass transfer occurs. Therefore, the high phenolic compound contents of the extracts obtained via DES-based UAE is considered to be due to the fact that the addition of water to DES lowered the viscosity, resulting in high diffusivity, and thus improved the extraction efficiency [27].

The IC_50_ values for the 1,1-diphenyl-2-picryl hydrazyl (DPPH) and 2,2′-azino-bis(3-ethylbenzothiazoline-6-sulfonic acid (ABTS) radical scavenging activities were 0.95 and 0.83 mg/mL in DES-based UAE and 1.31 and 1.82 mg/mL in HWE, respectively, indicating that the extract obtained via DES-based UAE had excellent antioxidant activity. Campos et al. [28] analyzed the phenolic compounds in an ethanol extract of maca root and reported that gallocatechin derivatives were present in the highest concentrations, ranging between 2.5 and 20.7 mg GAE/100 mL, followed by catechin derivatives, protocatechuic acid, and *p*-coumaric acid derivatives, which were between 1.4 and 15.2 and 0.6 and 0.4 mg GAE/100 mL, respectively. In addition, Sandoval et al. [29] reported that the catechin content in the water extract of maca root was 2.5 mg/g, so the results of this study showed that the catechin content of the maca leaf extract obtained using DES-based UAE was significantly higher. Catechin, a flavonol found in various foods such as wine, tea, and fruits, is recognized for its hydroxyl and peroxyl radicals, superoxide, DPPH radical scavenging activities, and iron-chelating effects [30]. Furthermore, gallic acid and its derivatives, which are widely distributed in the plant kingdom, exhibit various physiological activities, including neuroprotective effects, free radical scavenging, and anti-cancer properties [31,32]. The results above demonstrate that a combination of DES and UAE enhances the extraction yield of bioactive compounds such as saponins and polyphenols from maca leaves and increases the antioxidant activity of the extract. This is thought to be because DES-based UAE operates under milder conditions, requires less solvent, and improves mass transfer due to cell wall destruction via ultrasound. Therefore, DES-based UAE is considered to be an environmentally friendly and energy-efficient extraction method compared to conventional HWE and could be applied to extract heat-sensitive bioactive components from various plants.

## 3. Material and Methods

### 3.1. Plant Materials and Chemical Reagents

The maca plants used in this study were procured from the Youth Research Institute (Cheongdo, Republic of Korea). After removing the roots and washing the leaves, they were freeze-dried. The dried leaves were then pulverized using a food mixer (FM-681C, Hanil Electric Co., Ltd., Seoul, Republic of Korea) and passed through a 45-mesh screen (Chung Gye Indus, MFG, Co., Seoul, Republic of Korea). The resulting leaf powder was stored in a deep freezer (MDF, Sanyo Electric Co., Ltd., Osaka, Japan) at –40 °C until use.

Diaion HP resin, catechin, epicatechin, gallic acid, cinnamic acid, ferulic acid, caffeic acid, *p*-coumaric acid, protocatechuic acid, naringin, phosphoric acid, DPPH, and ABTS were purchased from Sigma-Aldrich (St. Louis, MO, USA). A Folin–Ciocâlteu phenol reagent and oleanolic acid were purchased from Junsei (Tokyo, Japan), and 100% acetonitrile was purchased from Burdick & Jackson™ (Honeywell International Inc., Charlotte, NC, USA). Choline chloride and glycerin was purchased from Daejung (Siheung, Korea), and all other chemicals used were of analytical grade.

### 3.2. Deep Eutectic Solvent Preparation

The DES for extracting bioactive compounds from maca leaves was prepared based on the results of a preliminary experiment conducted by referencing the method of Barbieri et al. [33] and Cai et al. [34]. That is, choline chloride and glycerin were mixed in a molar ratio of 1:2 and then stirred in a shaking water bath (BS-11, JeioTech Co., Ltd., Seoul, Republic of Korea) at 120 rpm at 80 °C until the color became transparent. After cooling the DES, its stability was checked by leaving it at room temperature overnight.

### 3.3. Optimization of Extraction Conditions

#### 3.3.1. Optimization Parameters Using Orthogonal Experiment Design

The optimization of DES-based UAE to extract the bioactive compounds from maca leaves was conducted according to the orthogonal experiment design of the Taguchi method [16]. Orthogonal matrix L_9_ (3^4^) was used, and four factors, liquid–solid ratio, water content added to DES, extraction time, and ultrasonic power, and three levels were set (Table 1). The extraction temperature was maintained at 38 ± 2 °C. To prevent an excessive increase in the extract temperature during extraction, a beaker containing the extract was placed in an ice bath and monitored using a temperature-sensing device. The DES-based UAE was performed according to each condition, and the response variables were TSC and TPC.

By applying Taguchi’s design to the experimental data, main effects plots were generated to identify the significant factors and optimize their levels to maximize the recovery of bioactive compounds. The S/N ratios were analyzed to determine the optimal conditions for each parameter, with the ‘larger is better’ criterion selected to maximize the extraction yield. Additionally, ANOVA was conducted to assess the influence of each parameter on the extraction responses and to evaluate the general linear regression model. The Taguchi experimental design and ANOVA were performed using Minitab^®^ 18 statistical software (Minitab Inc., State College, PA, USA).

#### 3.3.2. Determination of Total Saponin Content

The TSC of maca leaf extracts was determined using the vanillin-sulfuric acid method, as described by Lee and Chang [5]. For each concentration, 0.25 mL of the prepared extract sample was mixed with 0.25 mL of 8% (*w*/*v*) vanillin solution (in ethanol) and 2.5 mL of 72% sulfuric acid. The mixture was then incubated in a water bath at 60 °C for 10 min, followed by cooling in cold water for 5 min. Then, the absorbance was measured at 544 nm using a microplate reader (Epoch, BioTek Instrument Inc., Winooski, VT, USA). The TSC was calculated using a standard curve created with oleanolic acid solutions at different concentrations. The TSC was expressed as mg of OAE per gram of sample DW.

#### 3.3.3. Determination of Total Polyphenol Content

The TPC of the extract prepared from the maca leaves was determined using the method of Folin and Denis [35]. First, different concentrations of the extract were prepared. Then, 0.2 mL of Folin–Ciocâlteu’s phenol reagent was added to the test solutions, mixed thoroughly, and they were allowed to stand at room temperature for 3 min. Subsequently, 0.4 mL of 10% sodium carbonate (Na_2_CO_3_) and 4 mL of distilled water were added to the mixture, which was then left in a dark room at room temperature for 1 h. Then, the absorbance was measured at 720 nm using a microplate reader (Epoch, Bioteck Instrument Inc.). The TPC was quantified using a standard curve constructed with gallic acid solutions, and was expressed as mg GAE per gram of sample DW.

### 3.4. Comparison of Bioactive Properties of Maca Leaf Extracts

#### 3.4.1. Preparation of Maca Leaf Extracts

To obtain biocomponent-rich extracts from maca leaves, DES-based UAE was performed under the optimal conditions selected through the Taguchi method. That is, 5 g of maca leaves were mixed with 200 mL of DES containing 30% water, and extracted using an ultrasonicator (KFS-600N, Korprotech, Seoul, Korea) for 30 min. The ultrasonic power was 300 W, and the extraction temperature was maintained at 38 ± 2 °C. The extract was centrifuged at 4 °C and 9896× *g* for 20 min (Supra-21K, Hanil, Daejeon, Korea), and the supernatant was filtered to remove the DES with Diaion HP resin. The distilled water (9.5 mL) was added to a glass column (20 × 200 mm) equipped with a filter, and 25 mL of Diaion HP 20 resin was filled and left until the resin hardened. After 5 mL of the maca leaf extract was loaded, DES was removed with 30 mL of distilled water. Afterwards, 50 mL of 100% ethanol was added to elute the bioactive compounds. The elution process was repeated three times, and the collected fractions were freeze-dried and used as an extract for measuring bioactive properties.

To verify the efficiency of the optimized DES-based UAE approach for the extraction of bioactive compounds, HWE, a representative conventional extraction method, was performed for comparative purposes. That is, the sample (5 g) was mixed with 200 mL of distilled water and extracted using a shaking water bath (BS-11, JeioTech Daejeon, Korea) at 90 °C for 2 h. The supernatant obtained via centrifugation was freeze-dried and used as a comparative sample to measure the functional component content and antioxidant activity.

#### 3.4.2. Determination of Total Saponin and Polyphenol Contents

The TSC and TPC of maca leaf extracts obtained under optimal conditions were determined using the methods of Lee and Chang [5] and Folin and Denis [32], respectively, as described above. The TSC was expressed as mg of OAE and GAE per gram of extract powder, and TPC was expressed as mg GAE per gram of extract powder.

#### 3.4.3. Determination of Phenolic Compounds via HPLC

The phenolic compound content of maca leaf extracts obtained under optimized DES-based UAE conditions was measured using a high-performance liquid chromatography (HPLC) system (Waters 2695, Waters Corp., Milford, MA, USA) according to the method of Nour et al. [36], with modifications. The HPLC detection system utilized an ultraviolet detector (Waters 2489, Waters Corp.). The analytical column used was an Atlantis^®^ dC18 (4.6 × 150 mm, 5 μm, Waters Corp.); the column temperature was maintained at 34 °C. The mobile phase consisted of a gradient of 1% phosphoric and 100% acetonitrile. The injection volume was 10 μL, and the detection wavelength was set at 280 nm. The standards used for the calibration curve included catechin, epicatechin, gallic acid, cinnamic acid, ferulic acid, caffeic acid, p-coumaric acid, protocatechuic acid, and naringin. The quantity of the phenolic compounds in the extracts was calculated using the area under the calibration curve’s standard peaks.

#### 3.4.4. Measurement of Antioxidant Activity

The antioxidant activities of the maca leaf extracts obtained under optimal conditions were assessed using the DPPH and ABTS assays. Antioxidant activity was expressed as the half-maximal inhibitory concentration (IC_50_) values for the DPPH and ABTS radical scavenging activities.

The DPPH radical scavenging activity was determined using the method of Lee and Yoon [37], with modifications. An extract sample (100 μL) and 0.2 mM DPPH (200 μL) solution were added to a microplate, incubated at 37 °C for 30 min, and the absorbance was measured at 517 nm using a microplate reader (Epoch, BioTek Instruments Inc., Winooski, VT, USA). The DPPH radical scavenging ability was calculated using the following Equation (3):

DPPH radical scavenging activity (%) = (1 − (A − B)/C) × 100(3)

where A is the absorbance of the test sample, B is the absorbance of distilled water instead of DPPH, and C is the absorbance of distilled water instead of the test sample.

The ABTS radical scavenging activity was measured using the method of Lee and Yoon [38]. Initially, 7 mM ABTS and 2.45 mM potassium persulfate were dissolved in distilled water and kept in the dark for 12–14 h to form ABTS cation radicals (ABTS+). The resulting solution was then diluted with 80% ethanol to achieve an absorbance of 0.700 ± 0.002 at 734 nm. Subsequently, 15 μL of extract sample and 300 μL of diluted ABTS+ solution were added to a microplate and allowed to react for 6 min. The absorbance of the mixture was measured at 734 nm using a microplate reader (Epoch, BioTek Instruments Inc.) and the ABTS radical scavenging activity was calculated using Equation (4).

ABTS radical scavenging activity (%) = (1 − (A − B)/C) × 100(4)

where A is the absorbance of the test sample, B is the absorbance of distilled water instead of ABTS, and C is the absorbance of distilled water instead of the test sample.

### 3.5. Statistical Analysis for Validation

The validation experiments performed to confirm the extraction efficiency of the DES-based UAE method were carried out in triplicate, and the experimental results were expressed as the mean ± standard deviation. Significant differences between the DES-based UAE and HWE at a significance level of *p* < 0.05 were analyzed Via a *t*-test using SPSS (Ver. 23, IBM Corp., Armonk, NY, USA).

## 4. Conclusions

This study aimed to use Taguchi’s methodology to determine the optimal conditions for the DES-based UAE of bioactive compounds from maca leaves. Based on nine Taguchi design experiments, the optimal conditions for extracting saponins and polyphenols were determined as follows: 40 mL/g liquid–solid ratio, 30% water content in DES, 30 min extraction time, and 300 W ultrasonic power. Under optimized DES-based UAE conditions, the predicted values were 37.07 mg OAE/g and 6.82 mg GAE/g for the total saponin and polyphenol contents, respectively. The difference between the predicted and experimental values was minimal, indicating that the model was robust. Maca leaf extracts obtained under the optimized DES-based UAE conditions had higher saponins, polyphenols, phenolic compound contents, and antioxidant activities than were obtained Via HWE. In conclusion, DES-based UAE can be used as an efficient and economical alternative method for extracting bioactive compounds from maca leaves. Furthermore, the maca leaf extract obtained Via DES-based UAE offers potential applications as a functional food.

## Figures and Tables

**Table 1:** L_9_ orthogonal designs, levels of four factors, and experimental results.

Run No.	Factors				Responses		S/N Ratios	
	Liquid–Solid Ratio (mL/g)	Water Content (%)	Extraction Time (min)	Ultrasonic Power (W)	TSC (mg OAE/g DW)	TPC (mg GAE/g DW)	TSC	TPC
1	20	10	10	180	16.43 ± 0.28	3.12 ± 0.18	24.31	9.84
2	20	20	20	240	21.69 ± 1.27	3.10 ± 0.09	26.69	9.82
3	20	30	30	300	27.60 ± 0.11	4.43 ± 0.07	28.82	12.93
4	30	10	20	300	24.85 ± 0.60	4.74 ± 0.70	27.90	13.27
5	30	20	30	180	27.95 ±1.28	4.53 ± 0.27	28.91	13.08
6	30	30	10	240	30.26 ± 2.19	4.87 ± 0.74	29.56	13.50
7	40	10	30	240	27.84 ± 2.53	6.05 ± 0.17	28.80	15.63
8	40	20	10	300	31.98 ± 2.05	6.32 ± 0.32	30.05	15.99
9	40	30	20	180	33.43 ± 3.48	6.31 ± 0.24	30.37	15.99
*K*1 (TSC)	21.91	23.04	26.23	25.94				
*K*2 (TSC)	27.69	27.21	26.65	26.59				
*K*3 (TSC)	31.08	30.43	27.79	28.14				
Delta value (TSC)	9.17	7.39	1.56	2.20				
*K*1 (TPC)	3.55	4.64	4.77	4.65				
*K*2 (TPC)	4.71	4.65	4.72	4.67				
*K*3 (TPC)	6.23	5.21	5.00	5.16				
Delta value (TPC)	2.68	0.57	0.28	0.51				

TSC, total saponin content; OAE, oleanolic acid equivalent; TPC, total polyphenol content; GAE, gallic acid equivalent; DW, sample dry weight; and S/N, signal-to-noise. Experimental value is represented as mean ± standard deviation of triplicate. $K_{i}^{A}$ = (Ʃ response at A_i_)/3, where A is each factor-variable level. Delta value: Refers to the result of extreme analysis, Δ = max {$K_{i}^{A}$}−min{$K_{i}^{A}$}.

**Table 2:** Analysis of variance results of orthogonal design experiment on different extraction conditions of maca leaf saponins.

Factor	Degree of Freedom	Sum of Square	Mean of Square	F-Ratio	*p*-Value	Contribution (%)
Total saponin content						
Liquid–solid ratio	2	775.325	387.662	111.814	<0.001	51.85
Water content	2	494.352	247.176	71.294	<0.001	33.06
Extraction time	2	23.620	11.810	3.406	0.042	1.58
Ultrasonic power	2	46.080	23.040	6.646	0.003	3.08
Error	45	156.016	3.467			10.43
Total	53	1495.393				100
*R* ^2^	98.24%					
*R*^2^ (adj)	96.48%					
Total polyphenol content						
Liquid–solid ratio	2	65.018	32.509	218.623	<0.001	81.95
Water content	2	3.780	1.890	12.710	<0.001	4.76
Extraction time	2	0.837	0.418	2.813	0.071	1.06
Ultrasonic power	2	3.007	1.504	10.111	<0.001	3.79
Error	45	6.692	0.149			8.44
Total	53	79.334				100
*R* ^2^	96.89%					
*R*^2^(adj)	97.78%					

Contribution (%) = $\frac{Sum\;of\;square}{Total\;sum\;of\;square}\times 100$

**Table 3:** Confidence intervals and results of conformation experiments at optimal extraction conditions.

Response	Predicted Value	Predicted Confidence Interval at 95% Confidence Level	Experimental Value	Difference
Total saponin content (mg OAE/g DW)	37.07	34.64–39.49	38.02 ± 0.45	0.95
Total polyphenol content (mg GAE/g DW)	6.82	6.07–7.57	6.78 ± 0.12	0.04

OAE, oleanolic acid equivalent; GAE, gallic acid equivalent; and DW, sample dry weight. Experimental value is represented as mean ± standard deviation of triplicate.

**Table 4:** Comparison of bioactive compound contents and antioxidant activity of maca leaf extracts obtained via deep eutectic solvent-based ultrasound-assisted extraction and hot water extraction.

		DES-Based UAE	HWE
Total saponin content (mg OAE/g)		718.89 ± 0.95 ^*^	707.50 ± 2.18
Total polyphenol content (mg GAE/g)		260.19 ± 1.00 ^*^	224.90 ± 1.65
Phenolic compounds (mg/g)	Catechin	102.58 ± 1.93 ^*^	88.73 ± 0.25
	Epicatechin	7.96 ± 0.13 ^*^	7.29 ± 0.14
	Gallic acid	17.82 ± 0.32 ^*^	15.77 ± 0.25
	Cinnamic acid	8.24 ± 0.05 ^*^	6.89 ± 0.10
	Ferulic acid	6.96 ± 0.04 ^*^	5.25 ± 0.05
	Caffeic acid	3.89 ± 0.06 ^*^	3.61 ± 0.15
	*p*-Coumaric acid	1.22 ± 0.02 ^*^	0.97 ± 0.06
	Protocatechuic acid	6.92 ± 0.03 ^*^	5.43 ± 0.21
	Naringin	4.78 ± 0.06 ^*^	3.28 ± 0.11
IC_50_ value (mg/mL)	DPPH radical scavenging activity	0.95 ± 0.01 ^*^	1.15 ± 0.03
	ABTS radical scavenging activity	0.83 ± 0.04 ^*^	1.82 ± 0.03

DES, deep eutectic solvent; UAE, ultrasound-assisted extraction; HWE, hot water extraction; OAE, oleanolic acid equivalent; and GAE, gallic acid equivalent. Experimental value is represented as mean ± standard deviation of triplicate. ^*^ Significant differences between DES-based UAE and HWE extracts according to *t*-test (*p* < 0.05).

## Data Availability

The data are contained within the article.
